# The Acceptance of Cosmetic Surgery Scale: Confirmatory Factor Analyses and Validation among Serbian Adults

**DOI:** 10.1007/s12144-016-9458-7

**Published:** 2016-06-15

**Authors:** Marko Jovic, Marcos Sforza, Milan Jovanovic, Marija Jovic

**Affiliations:** 1 Clinic for Burns, Plastic and Reconstructive Surgery, Clinical Center of Serbia, Zvecanska 9, Belgrade, Serbia; 2 Dolan Park Hospital, Stoney Lane, B60 1LY, Bromsgrove, UK; 3 Department of Plastic and Reconstructive Surgery, School of Medicine, University of Belgrade, Zvecanska 9, Belgrade, Serbia; 4 Department of Marketing Management and Public Relations, Faculty of Organizational Sciences, University of Belgrade, Jove Ilica 154, Belgrade, Serbia

**Keywords:** Acceptance of cosmetic surgery scale, Validity, Confirmative factor analysis, Serbian adults

## Abstract

We examined the validity of the Serbian version of the Acceptance of Cosmetic Surgery Scale (ACSS; Henderson-King and Henderson-King 2005). A total of 622 Serbian adults completed the ACSS, along with Serbian translations of measures for the discrepancy between actual body weight and ideal body weight, body appreciation, sociocultural attitudes toward appearance, and demographics. Confirmatory factor analyses were conducted to compare how different ACSS models fitted the collected data. A three-factor model provided the best fit to the data relative to two- and one-factor models. The three-factor model had good internal consistency, convergent and discriminant validity, and nomological validity. The ACSS seems to be a valid instrument for use in Serbian populations. Our study will contribute towards better understanding of the acceptance of cosmetic surgery from a cross-cultural perspective.

## Introduction

Over recent decades, cosmetic surgery has become an important and challenging area in the continued expansion of plastic surgery. The American Society of Plastic Surgeons reported an increase of 111 % in cosmetic procedures from 2000 to 2014 (American Society of Plastic Surgeons 2015). Similarly, the International Society of Aesthetic Plastic Surgery recorded an increase of 84 % in cosmetic procedures undertaken by its members in South Korea from 2010 to 2014 (International Society of Aesthetic Plastic Surgery 2014).

This increase in the prevalence of cosmetic procedures suggests changes in people’s attitudes towards cosmetic surgery. Research in this area among Western populations appears to be well established and thriving (Henderson-King and Henderson-King 2005; Swami et al. 2009; Swami et al. 2011). However, little is known about attitudes toward cosmetic surgery among non-Western populations (Swami 2010). One of the possible reasons for insufficient understanding of non-Western population attitudes towards cosmetic surgery may come from the lack of reliable and valid translated scales for assessment of such attitudes (Swami 2010).

In studies conducted among Western populations, authors have mostly used the Acceptance of Cosmetic Surgery Scale (ACSS; Henderson-King and Henderson-King 2005). This scale is a multidimensional measure of various aspects of attitudes towards cosmetic surgery. ACSS is composed of three subscales, namely Intrapersonal, Social, and Consider. The Intrapersonal subscale measure attitudes related to the self-oriented benefits of cosmetic surgery. The Social subscale evaluates social motivations for cosmetic surgery. The Consider subscale measures the probability that a participant would consider having the cosmetic surgical procedure. The ACSS has been used among Western populations in North (Henderson-King and Brooks 2009; Menzel et al. 2011; Park et al. 2010) and South America (Carion et al. 2011; Neves et al. 2012; Swami et al. 2011), Europe (Stefanile et al. 2014; Swami and Hendrikse 2012; Swami et al. 2009), and Australia (Sharp et al. 2014; Slevec and Tiggemann 2010).

With regard to the latent structure of the ACSS, in their original work among adults living in the United States, Henderson-King and Henderson-King (2005) suggested a three-factor solution, but also noted that obtaining a total Acceptance score is acceptable. Among Western populations, the superiority of the three-factor solution for ACSS has been confirmed in a study based on confirmatory factor analyses (CFA) undertaken in Italian women (Stefanile et al. 2014). Similarly, the basic pattern of results was supported in Brazilian adults (Swami et al. 2011) after exploratory factor analyses (EFA).

Research using the ACSS appears to be extensive, but only few studies have administered the ACSS in non-Western populations (Swami 2010; Swami et al. 2012; Tam et al. 2012). Moreover, to the best of our knowledge, the factor structure of the ACSS has been examined only in Malaysia (Swami 2010) and South Korea (Swami et al. 2012). In these two studies, a two-factor solution was supported after EFA. In the Malay version of the ACSS, the first factor comprises the original Consider subscale while the second factor is represented by a combination of the original Intrapersonal and Social subscales (Swami 2010). Unlike previous work, in the South Korean version of the ACSS, the Consider subscale was different from the original one, as it included a number of items from both the Social subscale and the Intrapersonal subscale (Swami et al. 2012). Based on the high correlation between the two extracted factors and high internal consistency of an overall score of all 15 ACSS items, the authors suggest that the total Acceptance score be used in the Malay and South Korean context (Swami 2010; Swami et al. 2012). Among non-Western populations CFA have not been performed to corroborate the original structure of the ACSS.

As a contribution toward better cross-cultural understanding of attitudes towards cosmetic surgery, we investigated acceptance of cosmetic surgery and its correlates in a Serbian context. Serbia is not identified as part of “the West”, but is an example of a country where cultural influences mix, and where making the usual distinction between “Western” and “Eastern” cultural cores is not possible (Lazic 2003). Exploring populations with Western and Eastern cultural influences would be one way of extending findings previously published.

Focusing on non-Western populations is important to better understand the cross-cultural differences in attitudes toward cosmetic surgery and the reasons for considering cosmetic surgery (Swami et al. 2011). For example, Swami et al. (Swami 2010; Swami et al. 2012) noted that, among Eastern women, social reasons are as important as internal reasons with respect to acceptance of cosmetic surgery. This observation is in contrast to information from Western-based studies, whereby intrapersonal reasons tend to be dominant in regard to acceptance of cosmetic surgery (Henderson-King and Henderson-King 2005; Swami et al. 2011).

## The Present Study

The present study was undertaken to expand knowledge on attitudes towards cosmetic surgery. Our first aim was to examine attitudes towards cosmetic surgery among Serbian adults. Specifically, we evaluated the factor structure of the Serbian version of the ACSS. To accomplish this task, we investigated three models of the ACSS with factor structures from previous studies: (1) total ACSS, overall score of all 15 ACSS items (Henderson-King and Henderson-King 2005); (2) two-factor model, in which the first factor is the Consider subscale and the second factor is a compound of Intrapersonal and Social subscales (Swami 2010); and (3) the original three-factor model (Henderson-King and Henderson-King 2005). The second aim of the current work was to examine the reliability of the ACSS as well as convergent and discriminant validity.

We also aimed to examine the nomological validity of the scale in the Serbian context. Specifically, we evaluated the associations between the acceptance of cosmetic surgery and known predictors among Western and non-Western populations, namely discrepancy between actual body weight and ideal body weight, body appreciation, sociocultural attitudes toward appearance, and demographics (Swami 2010; Swami et al. 2011). We hypothesized that higher acceptance of cosmetic surgery scores would be positively correlated with weight discrepancy and sociocultural attitudes toward appearance and negatively correlated with body appreciation. Finally, we aimed to compare the acceptance of cosmetic surgery scores obtained in the Serbian context with those obtained in North America by Henderson-King and Henderson-King (2005) and in non-Western countries such as Malaysia (Swami 2010) and South Korea (Swami et al. 2012).

## Methods

### Participants

A total of 622 individuals (64.1 % women, *n* = 399; 35.9 % men, *n* = 223) aged 18–82 years (M = 42.3, SD = 14.4) were the study cohort. A total of 49.1 % of participants had been educated to graduate level, 44.3 % to secondary level, and 6.6 % to undergraduate level. The study comprised participants from inner-city areas (66.9 %), as well as those living in suburbs (24.9 %) or villages (8.2 %). Participants had a mean self-reported body mass index (BMI) of 24.34 (SD = 4.09).

### Measures

#### The Acceptance of Cosmetic Surgery Scale

(ACSS; Henderson-King and Henderson-King 2005). The 15-item ACSS. is a multidimensional measure of various aspects of attitudes toward cosmetic surgery. Three dimensions of such attitudes are measured: Intrapersonal (5 items; e.g., “Cosmetic surgery can be a big benefit to people’s self-image”), Social (5 items; e.g., “I would seriously consider having cosmetic surgery if my partner thought it was a good idea”), and Consider (5 items; e.g., “If I knew there would be no negative side effects or pain, I would like to try cosmetic surgery”). All items in the ACSS are rated on a seven-point Likert scale (1 = strongly disagree, 7 = strongly agree) and it has been shown to have high internal consistency, good test–retest reliability after three weeks, and good convergent and divergent reliability among Western samples (Henderson-King and Henderson-King 2005).

#### Photographic Figure Rating Scale

(PFRS; Swami et al. 2008). The PFRS is a measure of the discrepancy between actual body weight and ideal body weight. It consists of 10 greyscale photographic images of real women with different values for the BMI. Images are labelled with numbers from 1 to 10 (1 = lowest BMI; 10 = highest BMI). Only women were asked to complete the PFRS. They selected one photograph that best matches their current figure, and one that matches the figure that they would like to have. A measure was calculated by computing the absolute value of the difference between ideal and current ratings. Studies have shown that the PFRS retains cross-cultural validity (Swami et al. 2011), and that scores derived from the scale have high construct validity and good test–retest reliability after 3 weeks, and good construct validity (Swami et al. 2008).

#### Body Appreciation Scale

(BAS-2; Tylka and Wood-Barcalow 2015). The BAS-2 is a 10-item measure of positive body image. BAS-2 is a revised version of the original BAS (Avalos et al. 2005) as a result of development in the conceptual understanding of body appreciation (Swami and Ng 2015). Items are rated on a five-point Likert scale (1 = strongly disagree, 5 = strongly agree). Results from studies in the USA and Hong Kong have confirmed a one-dimensional-factor structure. Also, the BAS-2 has shown cross-cultural validity (Swami and Ng 2015), good test–retest reliability after 20 days, and good construct validity (Tylka and Wood-Barcalow 2015).

#### Sociocultural Attitudes Towards Appearance Questionnaire

(SATAQ-4; Schaefer et al. 2015). The SATAQ-4 is a 22-item measure of various societal and interpersonal aspects of appearance ideals. It represents an improved version of SATAQ-3 (Thompson et al. 2004), which was revised to provide for assessment of muscularity vs. thinness internalization, and indexes three domains of perceived socio-cultural pressures: media, family, and peers. Items are rated on a five-point Likert scale (1 = strongly disagree, 5 = strongly agree). The SATAQ-4 has been shown to consist of five subscales (Schaefer et al. 2015): two internalization subscales (Internalization – thin/low body fat; Internalization – muscular/athletic), and three pressure subscales (Pressure from media; Pressure from family; Pressure from peers). The Internalization – thin/low body fat subscale consists of five items that measure the ideal thinness. The Internalization – muscular/athletic subscale consists of five items that indicate endorsement and acceptance of the athletic physical ideal. Pressure subscales sought to assess one’s perception of receiving appearance-related pressures from peers (four items), family (four items), and the media (four items). Additionally, SATAQ-4 scale scores provided evidence for cross-cultural validity and good reliability and convergent validity (Llorente et al. 2015; Schaefer et al. 2015; Yamamiya et al. 2015).

#### Demographic Variables

Participants were asked to provide demographic data: sex, age, highest educational level, settlement type (inner-city area, suburb, village), self-reported height and weight. The latter two variables were used to calculate BMI (kg/m^2^).

### Procedure

The study protocol was approved by the Ethics Committee of the Medical Faculty in Belgrade, Serbia. Serbian versions of the ACSS, PFRS, BAS-2, and SATAQ-4 was developed initially using the standard back-translation method (Brislin 1970). Initially, we translated the scales into the Serbian language, and later this version was translated back into English by an independent translator. The two translators then corrected minor discrepancies for each of the scales. Data collection took place in two primary healthcare centers in Belgrade in 2015. Both centers were chosen randomly: one center from a city area (Health Center Savski Venac) and one from a mostly suburban area (Health Center Palilula). Participants were informed about the purpose of the research through the cover letter and, after ensuring anonymity, were given the questionnaire to complete. They participated voluntarily, were tested individually, and were not remunerated. The questionnaire took ≈15 min to complete.

### Statistical Analyses

A series of independent samples *t*-tests were used to ascertain if there were significant sex differences on ACSS items, ACSS subscales, and total ACSS scores. To account for multiple testing, we used the Bonferroni correction (Bonferroni 1936). The fit of the three ACSS models was estimated with CFA through LISREL v8.80 (Jöreskog and Sörbom 2006) starting from a matrix of polychoric correlations (Holgado-Tello et al. 2010; Jöreskog 1994) . Missing values were replaced with pattern-matching imputation using PRELIS (Jöreskog and Sörbom 1996). For data that did not follow a multivariate normal distribution, the Robust Maximum Likelihood method of estimation was used for CFA.

Fitness of the ACSS models was assessed using eight indices: (i) Satorra–Bentler Scaled Chi-Square (S-B χ2): ideally values should not be significant (Satorra and Bentler 2001); (ii) the ratio between S-B χ2 and degrees of freedom (S-B χ2/df): values <2 indicate a good fit, and values between 2 and 3 indicate an acceptable fit (Schermelleh-Engel et al. 2003); (iii) Root Mean Square Error Of Approximation (RMSEA): values of .08–.05 indicate an acceptable fit, and values <.05 indicate a good fit (Browne and Cudeck 1993); (iv) Parsimony Goodness of Fit Index (PGFI): optimal values are those near.5 (Mulaik et al. 1989); (v) Standardized Root Mean Square Residual (SRMR): values <.5 indicate a well-fitting model, and values <.10 are deemed acceptable (Byrne 1998); (vi) Non-Normed Fit Index (NNFI): values >.95 indicate an acceptable fit, and values >.97 suggest a good fit (Schermelleh-Engel et al. 2003); (vii) Comparative Fit Index (CFI): values >.95 indicate an acceptable fit, and values >.97 suggest a good fit (Schermelleh-Engel et al. 2003); (viii) Akaike’s Information Criterion (AIC): an estimation of the quality of each model, relative to each of the other models (Akaike 1973).

To determine internal consistency, Cronbach’s α value was calculated for each scale and its corresponding subscales. Cronbach’s α values >.70 are considered acceptable (Nunnally 1978). Convergent validity of the three models was assessed by examining factor loadings, average variance extracted (AVE) and composite reliability (CR) (Fornell and Larcker 1981). Models with an AVE > .5 and CR > .7 are considered compelling demonstration of convergent validity (Hair et al. 2009). Discriminant validity of three models was assessed by comparing the AVE values of each factor with the squared correlation between that factor and other factors in the model (Fornell and Larcker 1981). Models in which each factor had more internal variance than variance shared between factors were considered to meet the requirement of discriminant validity (Fornell and Larcker 1981).

To examine the factor structure of the BAS-2 and SATAQ-4, EFA were conducted using Varimax rotation. The number of factors to be extracted was determined by eigenvalues (λ > 1.0), inspection of Scree plots (Cattell 1966), the results of parallel analysis (Hayton et al. 2004), and extraction criteria of .40 (Kline 1986). A series of independent samples *t*-tests were used to ascertain if there were significant sex differences on BAS-2 scores as well as on SATAQ-4 subscales and to examine differences between Serbian, United States, Malay and South Korean participants on acceptance of cosmetic surgery scores. Nomological validity was examined by computing bivariate correlations between all of the ACSS subscales and PFRS, BAS-2, SATAQ-4 subscales, BMI, and participant age, separately for women and men. Correlations of .10 were considered to be “small”, correlations of .30 were considered to be “medium”, and correlations of .50 were considered to be “large” (Cohen 1992).

## Results

### Acceptance of Cosmetic Surgery

Descriptive statistics and mean comparisons between sexes (independent samples *t*-tests) for all ACSS items are presented in Table 1. Women had higher values for all ACSS items accept three items (# 9, 12, and 13). Due to Bonferroni correction, only those values of the *t*-test in which *p* < .003 were considered significant. For women, the highest score was for the fifth ACSS item (“If cosmetic surgery can make someone happier with the way they look, then they should try it”; M = 4.70, SD = 1.76), whereas the lowest score was for the thirteenth item (“ I would seriously consider having cosmetic surgery if I thought my partner would find me more attractive”; M = 2.32, SD = 1.73). For men, the highest score was for the fifth ACSS item (“If cosmetic surgery can make someone happier with the way they look, then they should try it”; M = 4.32, SD = 1.81), whereas the lowest score was for the ninth item (“I would seriously consider having cosmetic surgery if my partner thought it was a good idea”; M = 2.62, SD = 1.81).

**Table 1:** ACSS - descriptive statistics and mean comparisons across sexes

ACSS item	M	SD	Women		Men		Skewness	Kurtosis	t	Cohen d
			M	SD	M	SD				
Item 1	4.16	2.02	4.43	1.93	3.88	2.11	−.34	−1.16	3.27^**^	.27
Item 2	4.38	1.83	4.63	1.72	4.13	1.93	−.55	−.79	3.33^**^	.27
Item 3	3.06	1.92	3.29	1.99	2.82	1.84	.40	−1.13	2.90	.25
Item 4	4.13	1.89	4.34	1.89	3.91	1.88	−.34	−1.03	2.74	.23
Item 5	4.51	1.79	4.70	1.76	4.32	1.81	−.54	−.60	2.55	.21
Item 6	3.59	2.16	3.75	2.24	3.43	2.07	.12	−1.43	1.78	.15
Item 7	3.54	2.18	3.83	2.27	3.25	2.09	.14	−1.45	3.19^**^	.27
Item 8	2.89	2.05	3.10	2.15	2.67	1.94	.57	−1.11	2.44	.21
Item 9	2.49	1.80	2.35	1.78	2.62	1.81	.94	−.35	−1.83	.15
Item 10	4.06	2.13	4.21	2.10	3.90	2.16	−.10	−1.31	1.72	.14
Item 11	2.76	1.84	2.87	1.87	2.64	1.80	.61	−.90	1.54	.13
Item 12	2.91	1.86	2.84	1.88	2.97	1.84	.50	−1.05	−.79	.07
Item 13	2.54	1.77	2.32	1.73	2.76	1.80	.91	−.33	−2.97	.25
Item 14	3.54	1.93	3.65	1.93	3.42	1.93	.03	−1.26	1.43	.12
Item 15	3.25	2.03	3.37	2.14	3.13	1.92	.29	−1.32	1.40	.12
Total ACSS	3.45	1.50	3.58	1.53	3.32	1.46	.11	−.94	2.04^*^	.17

*ACSS* Acceptance of Cosmetic Surgery Scale; *n* = 622; *p < .05; **p < .003 due to Bonferroni correction

An independent samples *t*-test showed that women had significantly higher scores than men on the Intrapersonal subscale (women: M = 4.35, SD = 1.60; men: M = 3.93, SD = 1.66; t (620) = 3.08; *p* < .05; d = .26), Consider subscale (women: M = 3.64, SD = 1.85; men: M = 3.21, SD = 1.70; t (620) = 2.80; *p* < .05; d = .46), and total ACSS score (women: M = 3.58, SD = 1.53; men: M = 3.32, SD = 1.46; t (620) 2.04; *p* < .05; d = .17) and were not significantly different with regard to Social subscale (women: M = 2.75, SD = 1.59; men: M = 2.82, SD = 1.59; t (620) = .53; *p* = .59).

Data in Table 1 suggest that a problematic trend regarding normality was not detected. All values for univariate skewness were < 2, whereas all values for univariate kurtosis were < 7 (West et al. 1995). Mardia’s coefficient of multivariate kurtosis was 22.75, which is considerably greater than the critical value of 5 (Bentler 2006), suggesting that the distribution of the variables violated the assumption of multivariate normality.

**Fig. 1:**
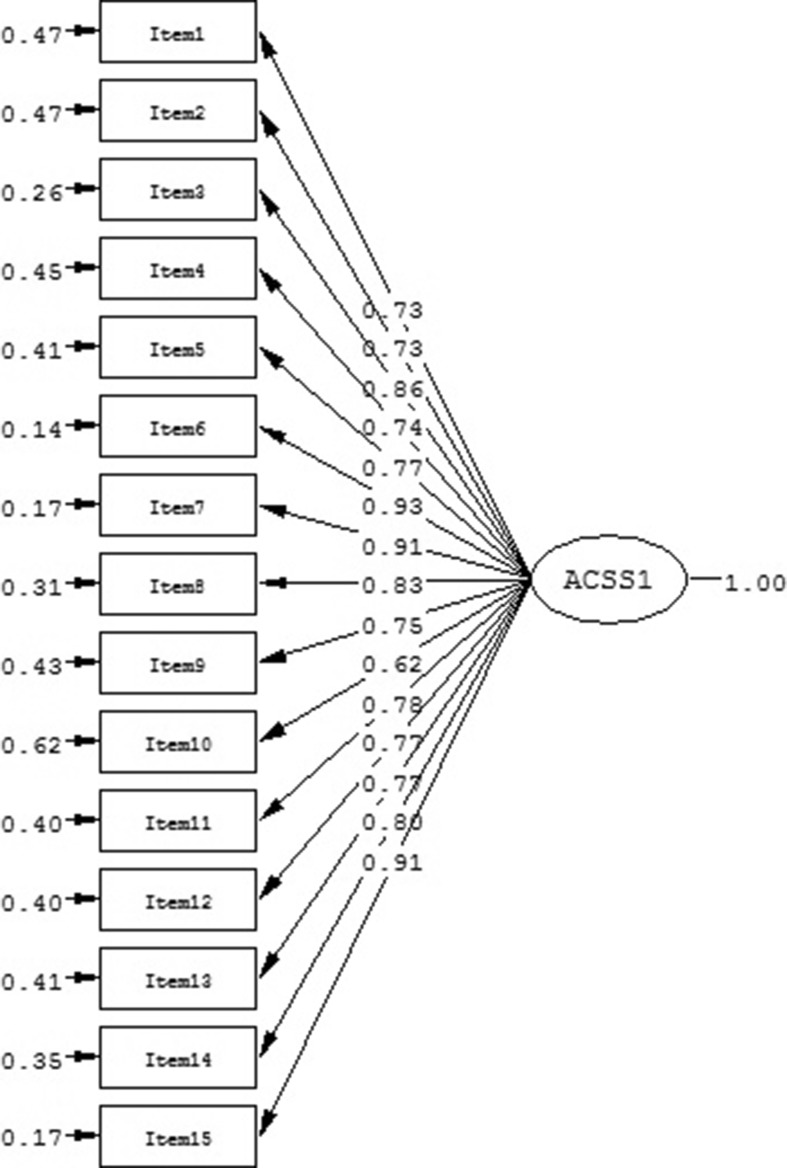
Standardized total ACSS model (model 1)

First, the fit of the total ACSS model (model 1) was examined (Table 2). The SRMR and the PGFI suggested a good fit, but the RMSEA, NNFI, and CFI did not. The S-B χ^2^ reached significance, which is to be expected in large samples (Browne and Cudeck 1993). Indices of fit suggested that model 1 provided only a marginally acceptable fit to the data. Standardized factor loadings (Figure 1) were all > .62 and were all significant (*p* < .01). Further, the total score of the Serbian ACSS showed high internal consistency (Cronbach’s α = .95) as well as convergent validity (CR = .96, AVE = .64 ).

**Table 2:** Confirmatory factor analyses of ACSS structural models (*N* = 622)

ACSS models	S-B χ^2^	df	SB χ^2^ / df	RMSEA	NNFI	CFI	SRMR	PGFI	AIC
Model 1	1437.28	90	15,97	.16	.94	.95	.08	.45	1497.28
Model 2	1282.79	89	14,41	.15	.95	.96	.08	.46	1344.79
Model 3	472.83	87	5,43	.08	.98	.99	.07	.59	538.83

*ACSS* Acceptance of Cosmetic Surgery Scale; S-B χ^2^ - Satorra-Bentler Scaled Chi-Square, *RMSEA* Root Mean Square Error of Approximation, *NNFI* Non-Normed Fit Index, *CFI* Comparative Fit Index, *SRMR* Standardized Root Mean Square Residual, *PGFI* Parsimony Goodness of Fit Index, *AIC* Akaike Information Criterion

**Fig. 2:**
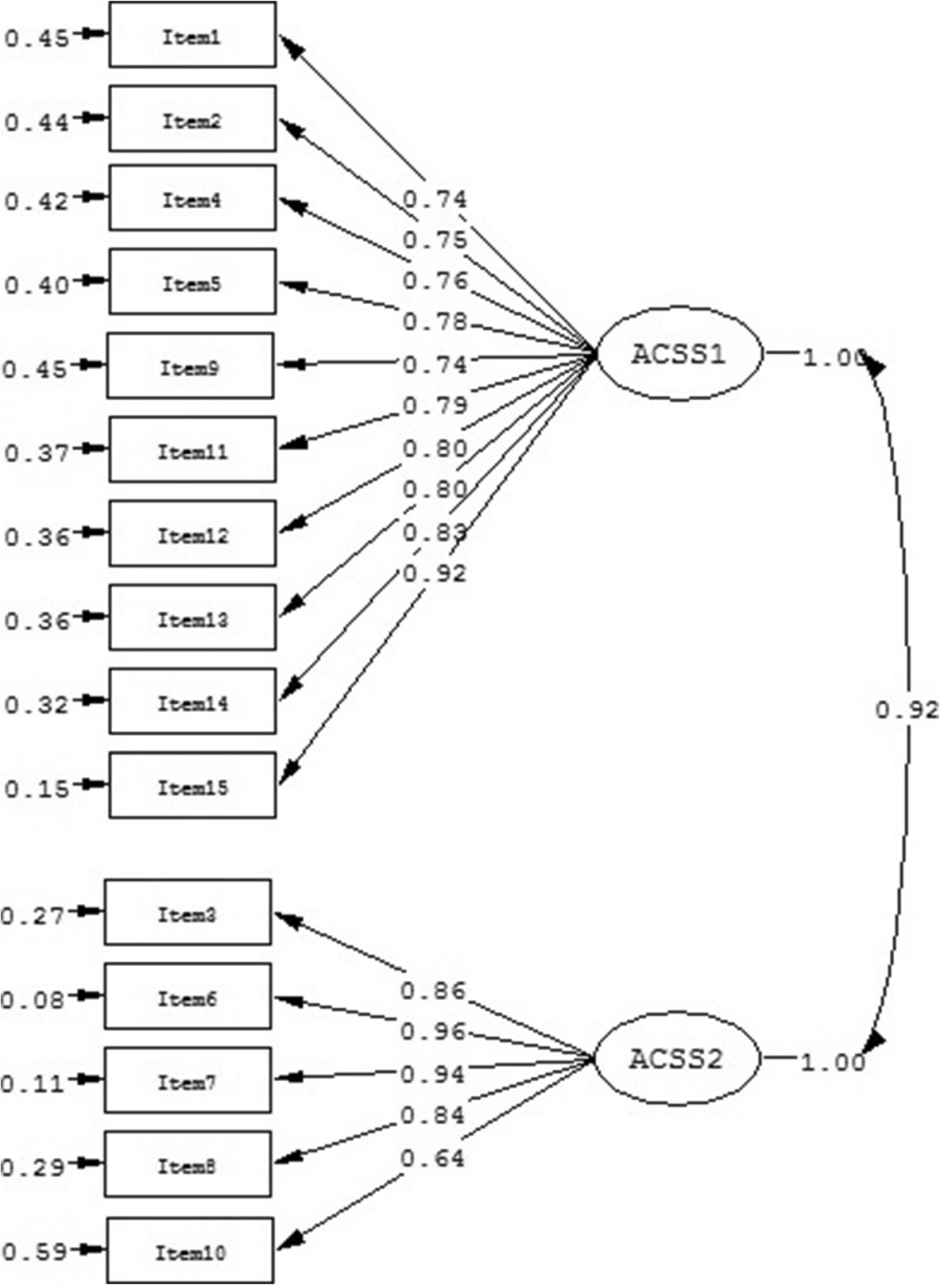
Standardized two-factor structure of the ACSS model (model 2)

Second, we fitted the two-factor model (model 2), in which the first factor is represented by a combination of Intrapersonal and Social subscales and the second factor comprises the Consider subscale. The NNFI, CFI, SRMR, and PGFI were within the acceptable range, but S-B χ^2^ reached significance and the S-B χ^2^/df was above the suggested level (Table 2). Indices of fit suggested that model 2 provided an only marginally acceptable fit to the data. Standardized factor loadings for this model were all significant (*p* < .01) and ranged from .64 to .96 (Fig. 2). These two factors were highly correlated (*r* = .81) and showed good internal reliability (Intrapersonal-Social: Cronbach’s α = .93 and Consider: Cronbach’s α = .91). Values of CR (Intrapersonal-Social: CR = .94 and Consider: CR = .93) and AVE (Intrapersonal-Social: AVE = .63 and Consider: AVE = .73) suggested good convergent validity for the model. The AVE of each factor was greater than the squared correlation between the two values showing good discriminant validity.

**Fig. 3:**
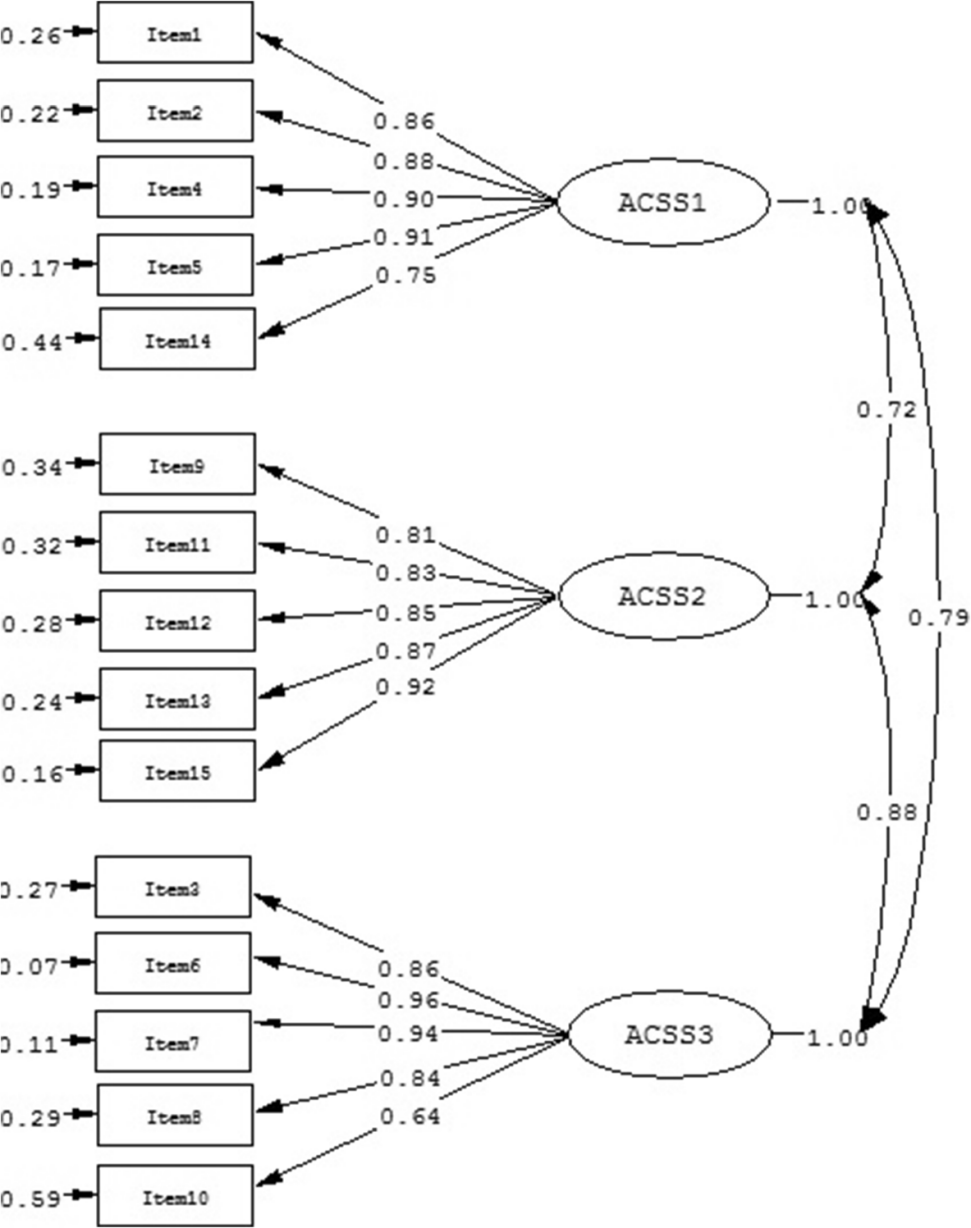
Standardized three-factor structure of the ACSS model (model 3)

Finally, we fitted the three-factor model of the ACSS (model 3). S-B χ^2^ reached significance and the S-B χ^2^/df value was above the suggested threshold of 3, but RMSEA showed an acceptable fit in this model (Table 2). Values of NNFI, CFI, SRMR, and PGFI showed a very good fit of model 3 to data. Standardized factor loadings were all significant (*p* < .01) and ranged from .64 to .96 (Fig. 3). All three factors were highly correlated with each other (Intrapersonal - Social: *r* = .64; Intrapersonal - Consider: *r* = .71; Social - Consider = .77; *p* < .01) and showed good internal reliability (Intrapersonal: Cronbach’s α = .92, Social: Cronbach’s α = .94, Consider: Cronbach’s α = .78). Convergent validity was good, showing high CR (Intrapersonal: CR = .93; Social: CR = .93; Consider: CR = .93) and high AVE (Intrapersonal: AVE = .74; Social: AVE = .63; Consider: AVE = .73) for all three factors. The AVE of each factor was greater than the squared correlations between them, and showed good discriminant validity.

Accordingly, model 3 provided the best fit in terms of all the indices of fit: S-B χ^2^, S-B χ^2^/df, NNFI, CFI, SRMR, PGFI, and SRMR. Model 3 was the only model in which RMSEA was within the range considered to be acceptable. Model 3 was more parsimonious than other models according to the lowest AIC value, thereby supporting the utility of the three-factor ACSS structure.

### Body Appreciation

The significance of Bartlett’s test of sphericity (χ^2^ (45) =4155.20, *p* < .01) and the size of the Kaiser–Meyer–Olkin measure of sampling adequacy (KMO = .95) showed that BAS-2 items had adequate common variance for EFA. Only one factor with an eigenvalue λ > 1.0 emerged from the analyses, thereby explaining 62.78 % of variance. The Serbian BAS-2 showed good internal consistency (Cronbach’s α = .93). An independent samples *t*-test showed that men and women differ significantly regarding body appreciation (women: M = 3.90, SD = .70; men: M = 3.75, SD = .79; t (620) = 2.56; *p* < .05).

### Sociocultural Attitudes towards Appearance

The significance of Bartlett’s test of sphericity (χ^2^ (231) = 12,273.53, *p* < .01) and the size of the Kaiser–Meyer–Olkin measure of sampling adequacy (KMO = .90) showed that SATAQ-4 items had adequate common variance for factor analyses. Five factors emerged from the analyses with an eigenvalue λ > 1.0. These factors reflected the original five SATAQ-4 factors delineated by Schaefer et al. (2015). Results of the Scree test and parallel analysis showed only four components with eigenvalues exceeding the corresponding criterion values for a randomly generated data matrix of identical size. Subsequently, it was decided to retain four factors for further investigation.

The four-factor solution (Table 3) explained 70.99 % of the variance, with factor 1 contributing 24.04 %, factor 2 contributing 17.60 %, factor 3 contributing 17.37 %, and factor 4 contributing 11.98 %. The first factor was a compound of the two original SATAQ-4 pressure subscales – Pressure from family and Pressure from peers – whereas the other factors were consistent with the original subscales –Pressure from media, Internalization muscular/athletic, and Internalization thin/low body fat (Schaefer et al. 2015). All four subscales showed good internal consistency (Factor 1: Cronbach’s α = .92, Factor 2: Cronbach’s α = .97, Factor 3: Cronbach’s α = .88, Factor 4 Cronbach’s α = .81).

**Table 3:** Factor loadings for the SATAQ-4

**SATAQ-4 item**	**Component**			
	**1**	**2**	**3**	**4**
12. I feel pressure from family members to improve my appearance	**.79**	.13	.04	.17
16. I feel pressure from my peers to improve my appearance	**.77**	.32	.32	−.06
13. Family members encourage me to decrease my level of body fat	**.77**	.11	−.12	.32
18. I get pressure from my peers to decrease my level of body fat	**.76**	.33	.28	.03
17. I feel pressure from my peers to look in better shape	**.76**	.33	.35	−.06
11. I feel pressure from family members to look thinner	**.75**	.13	.09	.26
15. My peers encourage me to get thinner	**.74**	.19	.17	.24
14. Family members encourage me to get in better shape	**.72**	.12	−.03	.24
21. I feel pressure from the media to improve my appearance	.22	**.93**	.07	.10
20. I feel pressure from the media to look thinner	.23	**.92**	.04	.17
22. I feel pressure from the media to decrease my level of body fat	.25	**.92**	.04	.17
19. I feel pressure from the media to look in better shape	.27	**.90**	.07	.10
2. I think a lot about looking muscular	.07	.04	**.81**	.14
6. I spend a lot of time doing things to look more athletic	.18	.05	**.78**	.26
1. It is important for me to look athletic	−.12	.05	**.77**	.16
7. I think a lot about looking athletic	.20	.10	**.76**	.35
10. I spend a lot of time doing things to look more muscular	.25	.00	**.72**	.21
5. I think a lot about looking thin	.35	.19	.20	**.67**
9. I think a lot about having very little body fat	.22	.07	.31	**.66**
4. I want my body to look like it has little fat	.04	.17	.17	**.64**
8. I want my body to look very lean	.33	.06	.37	**.58**
3. I want my body to look very thin	.15	.09	.43	**.57**

*SATAQ* Sociocultural Attitudes Towards Appearance Questionnaire

A series of independent samples *t*-tests showed that women and men were significantly different with regard to, Internalization of athletic/muscular look (women: M = 2.21, SD = .89; men: M = 2.58, SD = .96; t (620) = 4.74; *p* < .01; d = 40), Pressure from family and peers (women: M = 1.85, SD = .91; men: M = 2.03, SD = 1.0; t (620) = 2.30; *p* < .05; d = .19), and Pressures from media (women: M = 2.47, SD = 1.43; men: M = 2.19, SD = 1.31; t (620) = 2.42; *p* < .05; d = .20), and were not significantly different with regard to Internalization of thin/low body fat look (women: M = 2.48, SD = .90; men: M = 2.37, SD = .90; t (620) = 1.53; *p* = .13).

### Inter-Scale Correlation

Bivariate correlations between the three ACSS subscales and PFRS (women only, M = 1.38, SD = 1.19), BAS-2, four SATAQ-4 subscales, BMI, and participant age are presented separately for women and men in Table 4. In women, all of the three ACSS subscales were correlated significantly with ideal body weight–actual body weight discrepancy, BAS-2, all of the four SATAQ-4 subscales, and BMI. Intrapersonal and Social subscales had a significant correlation with participant age. In men, all of the three ACSS subscales were correlated significantly with BAS-2, as well as with all of the four SATAQ-4 subscales. ACSS subscales were not significantly correlated with BMI and age.

**Table 4:** Inter-scale correlations between ACSS subscales and all remaining variables^a^

	**1**	**2**	**3**	**4**	**5**	**6**	**7**	**8**	**9**	**10**	**11**
1. Intrapersonal		.67^**^	.76^**^	−.12^*^	.20^**^	.12^*^	.11^*^	.21^**^	.06	.08	.10^*^
2. Social	.62^**^		.78^**^	−.26^**^	.39^**^	.28^**^	.32^**^	.28^**^	.19^**^	.11^*^	.14^**^
3. Consider	.62^**^	.77^**^		−.19^**^	.32^**^	.25^**^	.24^**^	.25^**^	.15^**^	.13^**^	.07
4. General body appreciation	−.13^*^	−.14^*^	−.21^**^		−.20^**^	−.01	−.30^**^	−.19^**^	−.44^**^	−.34^**^	−.09
5. Internalization thin/low body fat	.17^*^	.34^**^	.27^**^	−.13^*^		.62^**^	.58^**^	.43^**^	.25^**^	.16^**^	−.02
6. Internalization athlete/muscular	.20^**^	.30^**^	.25^**^	.02	.61^**^		.35^**^	.20^**^	−.01	−.05	−.07
7. Pressure family and peers	.16^*^	.47^**^	.34^**^	−.23^**^	.48^**^	.33^**^		.49^**^	.48^**^	.48^**^	.08
8. Pressure media	.28^**^	.43^**^	.37^**^	−.29^**^	.30^**^	.25^**^	.58^**^		.21^**^	.23^**^	.04
9. Weight discrepancy	/	/	/	/	/	/	/	/		.71^**^	.15^**^
10. Body Mass Index	−.11	.03	.01	−.13	.02	−.14^*^	.23^**^	.15^*^	/		.18^**^
11. Age	.17^**^	.10	.05	−.02	.01	−.19^**^	.07	.11	/	.23^**^	

^a^Correlations for women in the top diagonal; women, *n* = 399; men, *n* = 223; ^*^ *p* < .05.; ^**^ *p* < .01

### Between-Study Differences

A series of independent samples *t*-tests showed that the Serbian sample (M = 3.45, SD = 1.50) had a significantly lower total Acceptance score than did the North American (M = 3.62, SD = 1.57, t (1303) = 1.99, *p* < .05, d = .11 ), Malaysian (M = 4.18, SD = 1.50, t (993) = 7.43, *p* < .01, d = .49), and South Korean (M = 4.18, SD = 2.58, t (887) = 5.28, *p* < .01, d = .34).

## Discussion

Results of the present study extend work examining the acceptance of cosmetic surgery by evaluation of the factor structure of the ACSS and its correlates among Serbian adults. Our results showed the superiority of the three-factor solution relative to two-factor solution and total Acceptance score after CFA. The three-factor solution showed good internal consistency, and provided good reliability of the Serbian ACSS. The ACSS also showed good convergent and discriminant validity. These findings suggest that among a Serbian-speaking population, it would be a desirable option to use the three ACSS subscales scores separately. Our results are in accordance with results among North American, Italian, and Brazilian adults (Henderson-King and Henderson-King 2005; Stefanile et al. 2014; Swami et al. 2011) that supported a three-factor solution. However, our results are not consistent with work among Malaysian (Swami 2010) and South Korean (Swami et al. 2012), which supported a two-factor solution following EFA. In accordance with the work of Henderson-King and Henderson-King (2005), all three ACSS subscales were highly inter-correlated, suggesting that the total ACSS score can also be used among Serbian population if it is a more preferable option.

Our results also showed that women have a significantly higher total ACSS score than that of men. These findings are in accordance with work done by Markey and Markey (2010) who found that women are more interested in cosmetic surgery than men. Likewise, other work has reported that women have greater acceptance of cosmetic surgery (Swami et al. 2009; Swami et al. 2012). As discussed by Brown et al. (2007), a possible cause for this sex difference comes from the greater sociocultural pressure on women to attain ideals of physical attractiveness. Other factors could influence greater acceptance of cosmetic surgery among Serbian women. For example, in Serbia, where cosmetic surgery is still in its infancy, cosmetic surgeons may target women specifically in their advertising, probably because women are the primary consumer group worldwide (International Society of Aesthetic Plastic Surgery 2014).

In addition to sociocultural-related causes, greater acceptance of cosmetic surgery among women in Serbia may be observed from an evolutionary perspective. Previous studies have shown that much of the motivation for women to improve their appearances may have evolutionary roots, rather than the social ones (Ferguson et al. 2011). According to Ferguson et al. (2011), female attractiveness is very important for both women and men. For women, the attractiveness is one of the key determinants of their mate value. At the same time, for men, female attractiveness is an indicator of the underlying reproductive value (Ferguson et al. 2011). Indeed, according to mate selection criteria in Serbia, thinness, attractiveness, good looks, and beauty are the traits that are most positively valued by men (Todosijević et al. 2003). This importance of female attractiveness could contribute to greater acceptance of cosmetic surgery among women than among men in Serbia.

Our findings suggest that the ACSS could be used for assessment of internal and external motivations for undergoing cosmetic surgery among a Serbian-speaking population. Specifically, in the present study, advantage was given to intrapersonal reasons over social reasons among women and men. Such findings are similar to research among Serbian women who reported internal rather than external reasons for undergoing breast-augmentation surgery (Nikolic et al. 2013). Likewise, among adults in the United States and Brazil, intrapersonal reasons have greater influence on acceptance of cosmetic surgery compared with social reasons (Henderson-King and Henderson-King 2005; Swami et al. 2011). However, among non-Western populations, intrapersonal and social reasons have equal influence on acceptance of cosmetic surgery (Swami 2010; Swami et al. 2012).

The present study also revealed good nomological validity of the Serbian ACSS. First, as predicted, we found significant correlations between higher scores of all of the three ACSS subscales with lower score of BAS-2, as well as with higher scores of all four SATAQ-4 subscales among women and men. Second, correlations between the three ACSS subscales and the actual–ideal body weight discrepancy as well as BMI were significant among women. Specifically, greater bias between the actual body figure and desired body figure as well as higher BMI increase acceptance of cosmetic surgery. These results are in accordance with work showing that acceptance of cosmetic surgery is correlated with actual body weight–ideal weight discrepancy (Swami 2010; Swami et al. 2011), body appreciation (Swami 2009, 2010; Swami et al. 2011; Swami et al. 2012), sociocultural attitudes towards appearance (Stefanile et al. 2014; Swami 2010; Swami et al. 2011; Swami et al. 2012), and BMI among women (Swami 2010; Swami et al. 2011). Our results are intriguing because they did not show a significant correlation between ACSS subscales and self-reported BMI among men. A possible explanation for these findings is that Serbian men may not perceive cosmetic surgery as a strategy to become thinner.

Results of the present study should be considered in relation to the Serbian versions of the SATAQ-4 and BAS-2. Our results showed that the Serbian SATAQ-4 was best reduced into a four-factor structure. Each of the four Serbian SATAQ-4 subscales showed good internal consistency. In addition, the one-dimensional-factor structure of the BAS-2 was confirmed, as reported among participants from the USA and Hong Kong (Swami and Ng 2015; Tylka and Wood-Barcalow 2015). The Serbian BAS-2 showed good internal consistency.

Finally, the overall ACSS scores in the present study were significantly lower than those reported in the United States (Henderson-King and Henderson-King 2005), Malaysia (Swami 2010), and South Korea (Swami et al. 2012). These findings might mirror the different prevalence of cosmetic procedures undergone by North Americans, Malaysians and South Koreans. Indeed, the United States ranks first in the world by number of both plastic surgeons and plastic surgery procedures (International Society of Aesthetic Plastic Surgery 2014), and Malaysia is a regional hub for medical tourism (Chaynee 2003), a large proportion of which includes cosmetic procedures. Similarly, rates of cosmetic surgery in South Korea have risen sharply over the past decade, mirroring rates in other East Asian nations (Kim 2003; McCurdy and Lam 2005). Although cosmetic surgery has recently become more popular in Serbia, we can suppose that Serbians are still more reticent than North Americans, Malaysians and South Koreans to accept these procedures.

Certain limitations of our results should be considered. First, the study sample was a consecutive series of participants attending primary-care settings (who could have different characteristics from those in the general population) during the observed period. Findings obtained in such specific settings cannot be easily generalized to the wider population. Furthermore, we did not assess the temporal stability of the ACSS. We evaluated the reliability of the ACSS only in terms of internal consistency. Finally, the present work included a limited number of scales that were developed initially in the West, and our translations of these scales may have been inadequate. Notwithstanding these limitations, the ACSS seems to be a useful measure of acceptance of cosmetic surgery among Serbian-speaking population.

The present study contributes to understanding of the attitudes towards cosmetic surgery from a cross-cultural perspective. The Serbian ACSS seems to be a valid and reliable instrument for measuring the extent to which Serbian adults are interested in undergoing cosmetic surgery by revealing consideration of cosmetic surgery as well as internal and external motivations. This study contributes toward better understanding of the growing interest in cosmetic surgery and the possible implications of such interest, in non-Western populations. Extending the availability of ACSS across new languages provides researchers with additional tolls for capturing the evolution of attitudes toward cosmetic surgery at the global level. The test–retest reliability of the ACSS will be a part of our future work in Serbia, along with examination of other factors that could influence acceptance of cosmetic surgery (e.g., personality, self-esteem).
